# Neuronal entry and high neurotoxicity of botulinum neurotoxin A require its N-terminal binding sub-domain

**DOI:** 10.1038/srep44474

**Published:** 2017-03-15

**Authors:** Jiafu Wang, Jianghui Meng, Marc Nugent, Minhong Tang, J. Oliver Dolly

**Affiliations:** 1 International Centre for Neurotherapeutics, Dublin City University, Glasnevin, Dublin 9, Ireland

## Abstract

Botulinum neurotoxins (BoNTs) are the most toxic proteins known, due to inhibiting the neuronal release of acetylcholine and causing flaccid paralysis. Most BoNT serotypes target neurons by binding to synaptic vesicle proteins and gangliosides via a C-terminal binding sub-domain (H_CC_). However, the role of their conserved N-terminal sub-domain (H_CN_) has not been established. Herein, we created a mutant form of recombinant BoNT/A lacking H_CN_ (rAΔH_CN_) and showed that the lethality of this mutant is reduced 3.3 × 10^4^-fold compared to wild-type BoNT/A. Accordingly, low concentrations of rAΔH_CN_ failed to bind either synaptic vesicle protein 2C or neurons, unlike the high-affinity neuronal binding obtained with ^125^I-BoNT/A (K_d_ = 0.46 nM). At a higher concentration, rAΔH_CN_ did bind to cultured sensory neurons and cluster on the surface, even after 24 h exposure. In contrast, BoNT/A became internalised and its light chain appeared associated with the plasmalemma, and partially co-localised with vesicle-associated membrane protein 2 in some vesicular compartments. We further found that a point mutation (W985L) within H_CN_ reduced the toxicity over 10-fold, while this mutant maintained the same level of binding to neurons as wild type BoNT/A, suggesting that H_CN_ makes additional contributions to productive internalization/translocation steps beyond binding to neurons.

Botulinum neurotoxins (BoNTs) are life-threatening proteins that potently and specifically bind to certain peripheral nerve endings and block the exocytotic release of transmitters. Exploiting their high specificity for cholinergic nerves, the large complex of BoNT/A containing haemagglutinin and other non-toxic proteins isolated from *Clostridium botulinum*, and to a lesser extent the type B counterpart, has proved successful in treating hyper-excitability disorders of muscles and secretory glands^1^. Also, the /A complex has been used for aesthetic/facial applications^2^,^3^. Furthermore, it benefits patients who suffer from certain types of migraine/headache^4^ due, at least in part, to blockade by BoNT/A or its complex of the exocytosis of substance P and calcitonin gene-related peptide from sensory fibres^5^,^6^,^7^. Recently, BoNT/A was reported to block tumour necrosis factor alpha (TNF-α) induced surface trafficking in sensory neurons of transient receptor potential (TRP) A1 and V1 channels; accordingly, enhancement by TNF-α of Ca^2+^ influx through these upregulated surface channels is abolished^8^.

All 7 serotypes of BoNTs (/A–/G) are mainly produced by the different types of *Clostridium botulinum* as single polypeptide chains (SC) (Mr ~ 150 k). Each is activated by *Clostridial* or host cell proteases to the highly-potent dichain (DC) form; this consists of an N-terminal ~50 k Zn^2+^-metalloprotease light chain (LC) linked to a 100 k heavy chain (HC) via a disulphide and non-covalent bonds. The crystal structures of BoNT/A, /B and /E have a tri-modular architecture^9^,^10^,^11^, with each serving a ‘chaperone-like’ role for the other domains. BoNT/A and /B are known to undergo acceptor-mediated endocytosis^12^, following binding to polysialo-gangliosides and synaptic vesicle proteins via the C-terminal half of HC (H_C_)^13^,^14^. Uptake of BoNT/A and /B into resting neurons^15^ requires lipid rafts, binding to their respective acceptors, synaptic vesicle protein 2 (SV2) and synaptotagamin, and passage through acidic compartments^16^. K^+^-depolarisation of cultured neurons recruits several endocytosis-promoting proteins (e.g. dynamin, clathrin, adaptor protein complex-2 and amphiphysin), thereby, enhancing toxin internalisation^16^. An acidic environment inside the vesicles induces the N-terminal half of HC (H_N_) to form a channel which allows the LC of each to unfold and cross the limiting membrane. In the cytosol, they regain enzymically-active structures and separate from their HC after reduction of the inter-chain disulphide^17^. Inhibition of thioredoxin reductase located on the synaptic vesicles prevents the paralysis induced by BoNTs^18^. The LCs cleave soluble N-ethylmaleimide-sensitive factor attachment protein receptors (SNAREs) [reviewed by refs 1 and 17]. The presence of a di-leucine motif in the LC of BoNT/A is responsible for it displaying the long-lasting duration of action in motor nerves^19^, due to persistently truncating synaptosomal-associated protein of 25 k (SNAP-25). This substrate is also susceptible to BoNT/E and /C1; the latter additionally truncates syntaxin, whereas BoNT/B, /D, /F and /G cleave vesicle-associated membrane proteins (VAMPs) at distinct bonds [reviewed in ref. 1]. Truncations of these SNARE proteins block fusion of synaptic vesicles and, hence, neurotransmitter release.

Significant advances have been made in identifying acceptors that bind to the C-terminal sub-domain of H_C_ (H_CC_) of BoNTs, and deciphering molecular details of their interactions. In addition to gangliosides, SV2 was discovered as an acceptor for /A, /D, /E and /F whereas synaptotagmin I and II serve this role for /B and /G [reviewed in refs 1 and 17]. Fibroblast growth factor receptor 3 (FGFR3) has also been reported to bind BoNT/A^20^. A high-resolution crystal structure of full-length BoNT/B complexed with the recognition domain of its acceptor revealed that the helix of synaptotagmin II binds to a saddle-shaped crevice at the C-terminal end of the H_CC_; this locus is adjacent to the non-overlapping ganglioside-binding site of BoNT/B^13^,^14^. Binding to the ganglioside GT1b also enables BoNT/B to sense low pH for directing formation of a translocation channel^21^; binding of both synaptotagmin II and gangliosides underlies its high selectivity and affinity. Recently, the crystal structures of H_C_ from BoNT/A bound to glycosylated and non-glycosylated SV2C-L4 were solved^22^,^23^. In addition to residues in H_CC_, several amino acids in H_CN_ were also reported to interact with N559 glycan in SV2C and contribute to binding to neurons^23^. It is noted that BoNT/D uses SV2s as receptors and enters neurons independently of the status of glycosylation of SV2^23^. This raises the possibility that H_CN_ may serve additional roles e.g. binding to unknown receptors, aiding internalisation and facilitating the channel formation for translocation of protease. H_CN_ is known to adopt a β-sheet jelly roll fold^24^, bind to micro-domains of the plasma membrane and interact with phosphatidylinositol phosphates^25^. The crystal structure has been reported of a BoNT mosaic serotype C/D with tetraethylene glycol (PG4)^26^, a moiety thought to mimic the hydrophobic fatty acid tails of phospholipids. Therefore, it was hypothesised that H_CN_ might be involved in interacting with phospholipid on the neuronal membrane^26^. Nevertheless, the functional role of H_CN_ in the multi-phasic action of BoNTs remains to be established.

Herein, the H_CN_ of BoNT/A is demonstrated to make an essential contribution to its extremely high lethality; the latter is dramatically decreased upon deleting this sub-domain. Recombinant BoNT/A devoid of the H_CN_ (rA∆H_CN_) virtually fails to truncate intra-neuronal SNAP-25. This is due to a lack of high-affinity (K_D_ = 0.46 nM) binding to neurons seen with ^125^I-radiolabelled BoNT/A. Although a higher concentration of rA∆H_CN_ did interact with neurons, the majority failed to get internalised as revealed using novel engineered tagged-derivatives. Furthermore, full-length BoNT/A containing a mutated H_CN_ residue (Trp 985) retains high-affinity neuronal binding but exhibits significantly reduced ability to cleave intra-neuronal target. Therefore, we have deduced that H_CN_ is involved in binding to neurons and entry of BoNT/A leading to proteolytic inactivation of SNAP-25.

## Results

### Recombinantly-produced BoNT/A (rA) with its H_CN_ sub-domain deleted exhibits unaltered protease activity

**Figure 1:**
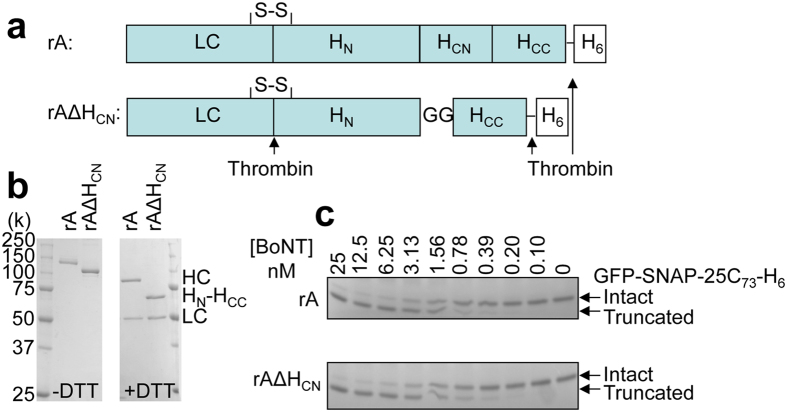
Expressed, purified rAΔH_CN_ and rA showed similar proteolytic activity. (**a**) Schematic of rA and rAΔH_CN_. Nucleotides encoding I_874_-Q_1091_were deleted and replaced with 6 nucleotides GGCGGT to generate rAΔH_CN_. S-S denotes inter-chain disulphide, GG between H_N_ and H_CC_ in rAΔH_CN_ represent glygly residues. H_6_ indicates 6xHis and (↑) represent consensus site for thrombin. (**b**) rAΔH_CN_ and rA expressed in *E. coli*, purified and nicked before being subjected to SDS-PAGE in the presence or absence of DTT, followed by Coomassie staining. Note that HC and H_N_-H_CC_ were separated from LCs only under reducing conditions. (**c**) rAΔH_CN_ and rA exhibited similar protease activity towards a model GFP-SNAP-25C_73_-His_6_ substrate.

To investigate the role of H_CN_, nucleotides encoding residues (I874-Q1091) were first deleted (Fig. 1a) from a pET29a-BoNT/A gene construct, previously described^19^. The resultant construct was transformed into *E. coli* for expression, by auto-induction. The deleted variant protein (rAΔH_CN_) was fully purified as a SC protein using immobilised metal affinity chromatography (IMAC), followed by anion-exchange chromatography as for rA (Fig. S1). Note there was no significant difference in terms of yield (~5 mg/L culture) and purity between rA∆H_CN_ and rA. After incubation with thrombin, the rA∆H_CN_ SC was converted to a DC form with an expected Mr (~125 k); its constituent LC (~50 k) and H_N_-H_CC_ (~75 k) were separated by SDS-PAGE only in the presence of reducing agent, confirming that the inter-chain disulphide had been formed in the expressed protein (Fig. 1b). Retention by rAΔH_CN_ of proteolytic activity was confirmed towards a recombinant model substrate (GFP-SNAP-25C_73_-His_6_)^27^,^28^ showing a similar EC_50_ value to rA (Fig. 1c). Therefore, it is clear that deleting H_CN_ from BoNT/A does not affect the enzyme activity of its integral LC.

### Deletion of H_CN_ from BoNT/A significantly decreases its intra-neuronal cleavage of SNAP-25 and lethality

**Figure 2:**
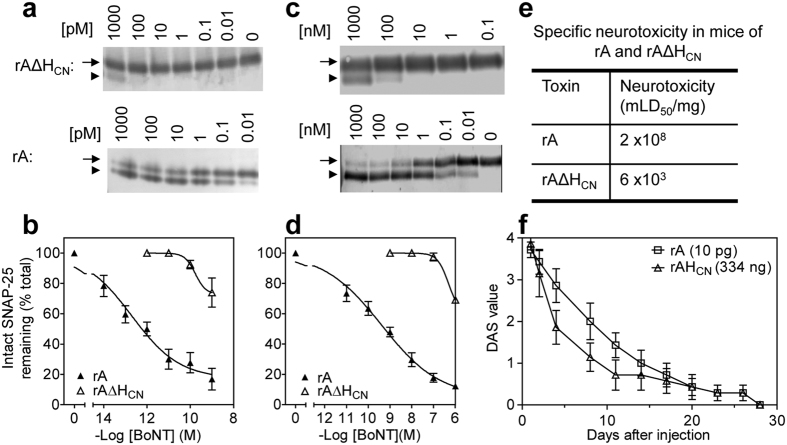
rAΔH_CN_ caused minimal cleavage of neuronal SNAP-25 and displayed greatly reduced lethality. Rat cultured CGNs (**a**,**b**) or TGNs (**c**,**d**) were incubated with various concentrations of rA or rAΔH_CN_ in medium for 24 h before harvesting in LDS sample buffer. Samples were separated by SDS-PAGE followed by Western blotting with an antibody recognising both intact and cleaved SNAP-25. The arrow and arrowhead in panel a and c indicate intact and cleaved SNAP-25, respectively. Data are mean ± S.E.M, n = 3. (**e**) Removal of the H_CN_ sub-domain from rA dramatically reduced its lethality in mice. (**f**) DAS values were recorded over 28 days after injecting rA or rAΔH_CN_ into the right gastrocnemius muscle of mice; SEM values are shown from 7 mice for each toxin. Two-way ANOVA with Bonferroni post hoc test analysis highlights that there is no significant difference (p > 0.05 at all time points) between the curves for rA and a 33,400-fold higher quantity of rAΔH_CN_.

The combined functional properties of rAΔH_CN_ were examined following exposure to rat cultured cerebellar granule neurons (CGNs), with subsequent monitoring of SNAP-25 truncation. This reflects the extent of internalisation and translocation of LC into cytosol where it acts on its target. Overnight incubation of 1 nM rAΔH_CN_ with the cultured neurons resulted in only a small fraction of SNAP-25 being cleaved, which equates to the amount produced by as little as 0.1 pM of wild-type (WT) rA (Fig. 2a). Extrapolation of this data revealed that deleting H_CN_ caused ~10^4^-fold drop in SNAP-25 cleavage (Fig. 2b). Similar results were obtained upon comparing SNAP-25 cleavage by rA and rAΔH_CN_ after overnight exposure to rat cultured trigeminal ganglion neurons (TGNs); again, deletion of H_CN_ resulted in ~10^4^-fold decrease in the degree of SNAP-25 cleavage (Fig. 2c and d). As rAΔH_CN_ and rA displayed similar protease activity *in vitro* towards a recombinant substrate (cf. Fig. 1c), it is reasonable to suspect that this dramatic decrease in SNAP-25 cleavage arose from defective internalisation and/or translocation of LC to the cytosol. The *in vitro* data concur with the results observed *in vivo*; intra-peritoneal injection of as much as 167 ng of rAΔH_CN_ was needed to kill 50% of the mice, corresponding to a toxicity of 6 × 10^3^ lethal doses (LD_50_)/mg. This is ~3.3 × 10^4^-fold lower than that for its WT (Fig. 2e). Thus, it was necessary to inject a ~33,400-fold larger quantity of rAΔH_CN_ than rA to induce muscle weakening to a similar extent (Fig. 2f); administering 10 or 1 ng of rAΔH_CN_ failed (data not shown) to induce a measurable digit abduction score (DAS)^29^. Our collective findings highlight the dramatic loss in the overall biological activity upon deleting the H_CN_ from rA.

### Low concentrations of rAΔH_CN_ fail to bind SV2C and intact neurons

**Figure 3:**
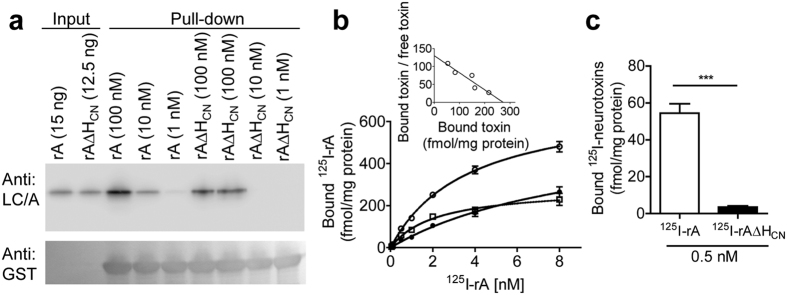
Deletion of H_CN_ from rA diminished its binding to SV2C-L4 and intact neurons. (**a**) Western blot from the *in vitro* pull-down assay (see Materials and Methods) shows that rAΔH_CN_ at 10 and 1 nM concentrations failed to bind non-glycosylated GST-SV2C-L4, in contrast to rA. At 100 nM, a lower amount of rAΔH_CN_ than rA was retained by immobilised SV2C-L4. Note that samples were reduced by DTT before SDS-PAGE. (**b**) CGNs were incubated with increasing concentrations of ^125^I-rA alone (○) or with 1 μM BoTIM/A (•) for 1 h at 4 °C followed by three washes before γ counting of the pellets. Subtracting non-saturable binding (•) from the total binding (○) yielded the saturable component (□). Inset: Scatchard plot of the saturable binding of ^125^I-BoNT/A to CGNs. (**c**) Binding of ^125^I-labelled rAΔH_CN_ to CGNs, measured as in (**b**) was non-significant. Data are mean ± S.E.M from two independent experiments performed in duplicates.

To decipher the detrimental effects of deleting H_CN_, the first step of the intoxication process was examined. For assessing whether deletion of H_CN_ from rA alters its binding to the known protein acceptor, a well-established albeit qualitative pull-down assay was performed, using immobilised the bacterially-expressed fourth loop (L4) of SV2C (residues 454–580) fused to glutathione S-transferase (GST)^30^,^31^,^32^. rAΔH_CN_ gave undetectable binding to this non-glycosylated SV2C at 1 or 10 nM, unlike rA (Fig. 3a). In contrast, binding of a high concentration of rAΔH_CN_ (100 nM) was observed, but at reduced levels compared to rA (Fig. 3a). For more in-depth analysis of the binding of rA and rAΔH_CN_ to CGNs, a sensitive and quantitative isotope assay was employed. rA and rAΔH_CN_ were labelled with [^125^I]iodine to high specific activities (920 and 840 Ci/mmol, respectively), using an established chloramine-T method^33^. Incubation of increasing concentrations of ^125^I-rA with CGNs revealed that the level of saturable binding reached a plateau at 8 nM, which was calculated by subtracting non-saturable binding in the presence of 1 μM unlabelled protease-inactive form of BoNT/A (BoTIMA)^19^ from the total binding (Fig. 3b). Scatchard analysis (Fig. 3b inset) revealed high-affinity binding of ^125^I-rA to CGNs (*K*_d_ = 0.46 ± 0.03 nM from two independent experiments), which is similar to the value of ^125^I-labelled native BoNT/A binding to rat brain synaptosomal membranes^33^. Notably, deletion of H_CN_ from ^125^I-rA drastically reduced its ability to bind to CGNs (Fig. 3c).

### A high concentration of rAΔH_CN_ binds but fails to enter into neurons

**Figure 4:**
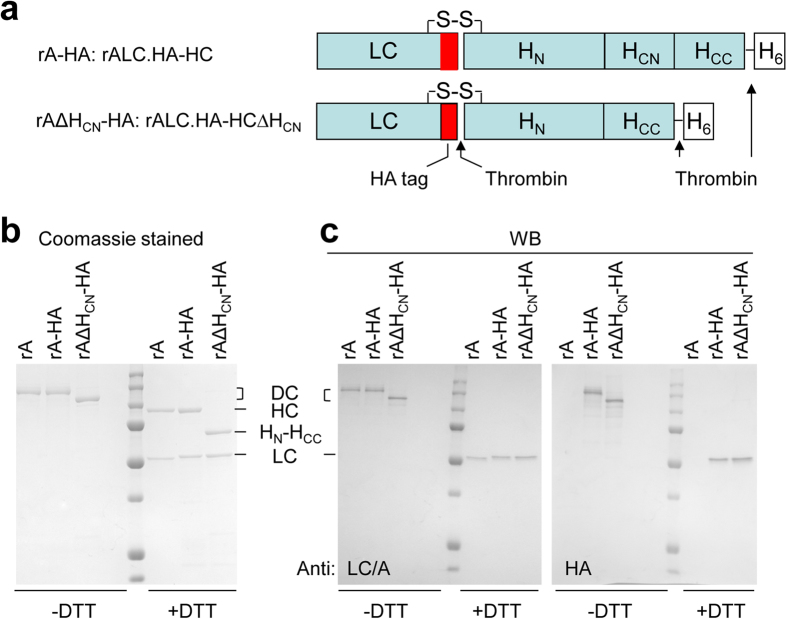
Generation of probes for visualising the cellular location of rAΔH_CN_ and rA. (**a**) Schematic of the BoNT/A probes generated. A short length of nucleotides encoding the HA tag was inserted before the thrombin cleavage site in the loop region of rA and rAΔH_CN_ to yield constructs encoding rA LC.HA-HC and rA LC.HA-H_N_H_CC_. For simplicity, the latter two were termed rA-HA and rAΔH_CN_-HA, respectively. After purification, nicked rA-HA and rAΔH_CN_-HA were subjected to SDS-PAGE in the presence or absence of DTT, followed by Coomassie staining (**b**) and Western blotting, using the indicated antibodies (**c**). rA was loaded for comparison. HA antibody only recognised LCs containing the HA tag. Note that due to the insertion of HA tag, LC-HA migrated slightly slower than WT LC.

**Figure 5:**
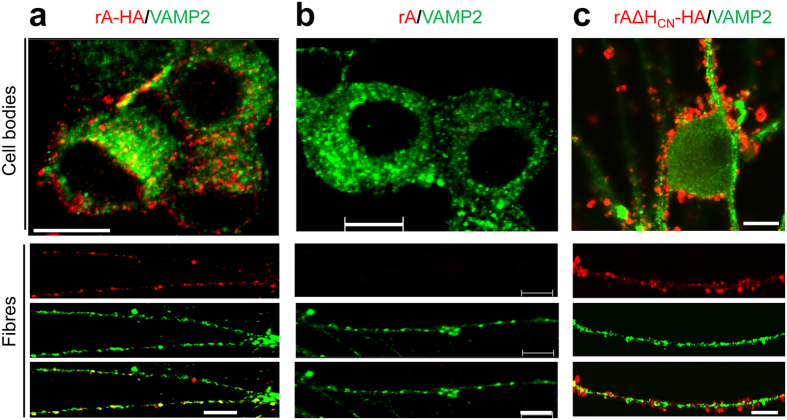
A majority of rAΔH_CN_-HA failed to enter cultured TGNs unlike rA-HA. Rat TGNs on coverslips were incubated with 100 nM of rA-HA (**a**), rA (**b**), or rAΔH_CN_-HA (**c**) for 24 h at 37 °C in culture medium. Washed cells were fixed with paraformaldehyde, permeabilised and blocked with BSA. Paired primary antibodies [rabbit monoclonal anti-HA and mouse monoclonal anti-VAMP2] were added for 1 h. Washed samples were incubated with Alexa Fluor 488 goat anti mouse IgG and Alexa Fluor 568 goat anti-rabbit IgG for 1 h. Images of cell bodies and fibres were captured with a confocal microscope. Representative images from three independent experiments are shown. Bars, 10 μm.

In addition to the above-noted absence of high-affinity binding upon deleting rAΔH_CN_, it was pertinent to establish whether this deletion inhibited internalisation of toxin bound at a higher concentration. To permit monitoring of the trafficking, tagged variants were engineered by incorporating a haemagglutinin (HA) epitope before the thrombin recognition sequence in the loop region of rA and rAΔH_CN_ (Fig. 4a). This allowed their respective locations to be visualised by means of a commercially-available antibody for recognising HA. Incorporation of HA did not affect the expression pattern, purity (Fig. 4b), yield or protease activity of either rA or rAΔH_CN_ (data not shown). As expected, anti-HA antibody only recognised the LCs with HA tag in rA-HA and rAΔH_CN_-HA but not the wild-type (WT) LC in rA (Fig. 4c). The larger size of TGNs (soma diameter ~25 μm) compared to that of CGNs (~6 μm) facilitated more clear-cut cellular localization of the tagged toxins. Immuno-cytochemistry and confocal microscopy of cultured TGNs, incubated for 24 h with 100 nM rA-HA followed by fixation and permeabilisation, revealed labelling associated with the plasma membrane (possibly the inner side) and some vesicular regions in the cell body (Fig. 5a). Punctate staining was also observed along the neurites, to some extents co-localised with VAMP2 (Fig. 5a), a vesicle marker. Again, this HA antibody could not visualise WT BoNT/A devoid of the tag (Fig. 5b), confirming its specificity. A majority of rAΔH_CN_ appeared clustered at the plasma membrane, and on neurites, even after 24 h incubation (Fig. 5c and Fig. S2). Similar experiments repeated except omitting permeabilisation indicated punctate labelling with rAΔH_CN_-HA on the outer surface of cell body and neurites, revealed using anti-HA antibody (Fig. S2). Moreover, TGNs incubated with 100 nM rAΔH_CN_-HA and an excess (1 μM) of non-tagged rAΔH_CN_ in high (60 mM) K^+^ (HK) buffer^16^ for 10 min exhibited reduced labelling of neurites compared to that treated with tagged toxin only (Fig. S3). Thus, rAΔH_CN_ seems unable to enter neurons even when a high concentration had to be employed to achieve binding.

### Tryptophan 985 in the H_CN_ of BoNT/A contributes to its neuronal internalisation/protease translocation steps

**Figure 6:**
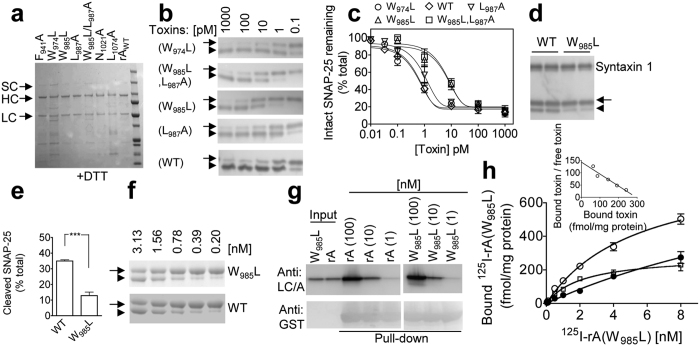
Effects of mutations in the H_CN_ of BoNT/A on its multi-functional activities in rat CGNs as monitored by intracellular cleavage of SNAP-25. (**a**) The expressed and purified mutants were nicked and subjected to SDS-PAGE and Coomassie staining in the presence of DTT; note that thrombin converted the majority of the SC to DC forms. (**b**) After 24 h exposure of CGNs to the different concentrations of the toxin variants in culture medium, the samples were subjected to SDS-PAGE followed by Western blotting. (**c**) Extents of SNAP-25 cleavage in CGNs by W_985_L and W_985_L/L_987_A were decreased > 10-fold compared to that of rA WT. Changing W_974_ to L did not affect the functioning of BoNT/A, whereas mutant L_987_A exhibited a mild drop in its activity. Note that in some cases error bars are encompassed by symbols. (**d**,**e**) CGNs were treated with 0.5 nM W_985_L or its WT in 5 mM K^+^ (LK) buffer for 8 min at 37 °C. Unbound toxin was removed by three washes before incubation in medium for 5 h to allow internalised toxin to cleave SNAP-25. The arrow and arrowhead in panel b and d indicate intact and cleaved SNAP-25, respectively. Data in panel c and e are mean ± S.E.M, n = 3. ***P < 0.001. (**f**) A representative protein stained gel showing BoNT/A WT and W_985_L mutant have similar protease activities in cleaving GFP-SNAP-25C_73_-His_6_ substrate. The arrow and arrowhead indicate intact and cleaved substrate, respectively. (**g**) Non-glycosylated GST-SV2C pulled down BoNT/A WT and W_985_L variant to similar extents, revealed by Western blotting using antibodies indicated. (**h**) Binding of ^125^I-rA(W_985_L) to CGNs was performed as in Fig. 3b. Subtracting non-saturable binding in the presence of 1 μM unlabelled rA(W_985_L) SC (•) from the total (○) yielded the saturable values (□). Inset: Scatchard plot analysis. Data are mean ± S.E.M from two independent experiments performed in duplicates.

In search of key residues in H_CN_ of full-length BoNT/A which might contribute to entry resulting from its binding to acceptor, residues thought pertinent to PG4 interaction (see Introduction) were mutated. A series of 8 constructs were made containing 1 or 2 mutations such as F_941_A, W_974_L, W_985_L, W985F, L_987_A, W_985_L/L_987_A, N_1021_A or L_1074_A. The resultant constructs were transformed into *E. coli* for expression; IMAC was used to purify the recombinant mutated and WT proteins. Curiously, no intact protein was obtained for the W985F mutant. Incubation of other mutants or WT with thrombin converted a majority of the SC to the DC form (Fig. 6a). The final yield of single (W_974_L) or double (W_985_L/L_987_A) mutants was decreased by ~10- and 5-fold compared to WT, respectively, whereas the others expressed to levels similar to that of the WT. For standardised measurement of the functionality of each partially purified mutant, their concentrations were adjusted according to that of intact DC rather than total concentration, using WT DC as reference. Initial attempts made to assess if PG4 binds rA proved negative, using a dot blot assay (Fig. S4). Likewise, pre-incubation of 100 pM rA with 1 μM PG4 did not affect subsequent cleavage of intra-neuronal SNAP-25 (Fig. S4).

To evaluate the multiple activities (e.g. acceptor binding/internalisation/cytosolic translocation i.e SNAP-25 cleavage) of the mutants, CGNs were cultured and incubated with WT or each variant at various doses in culture medium for 24 h at 37 °C. This revealed a mild drop in activity of mutant L_987_A (Fig. 6b,c). Changing W_974_ to L did not seem to affect the overall functioning of BoNT/A (dose response curve is near identical to WT: Fig. 6c). Similarly, mutating N_1021_ to A failed to alter SNAP-25 cleavage whereas mutant F_941_A and L_1074_A exhibited minimal decreases in their activities (Fig. S5). The cleavage of SNAP-25 in CGNs dropped over 10-fold after a single (W_985_ to L) or double mutation (W_985_ to L and L_987_ to A) (Fig. 6b,c). The importance of W_985_ for the action of BoNT/A was further investigated by incubating rCGNs with W_985_L mutant or WT for 8 min in low (5 mM) K^+^ buffer^16^; then, the cells were washed three times to remove unbound toxin and further cultured for 5 h. This mutant gave significant decrease in cleavage of SNAP-25 compared to WT (Fig. 6d,e), but the mutation did not affect its *in vitro* protease activity (Fig. 6f) or binding to non-glycosylated SV2C (Fig. 6g). To investigate whether mutating W985 affects its binding to acceptors on CGNs, this mutant was labelled with ^125^I to specific activity ~740 Ci/mmol. Notably, ^125^I-rA(W_985_L) showed near identical binding affinity (*K*_d_ = 0.47 ± 0.01 nM from two experiments) (Fig. 6h and inset) as ^125^I-labelled rA (cf. Fig. 3b). Hence, it is reasonable to deduce that W985 in BoNT/A plays some part in its neuronal internalisation and/or protease translocation after initial binding.

## Discussion

It is reported herein that BoNT/A lacking the H_CN_ sub-domain exhibits greatly reduced activity in cleaving intra-neuronal SNAP-25, and dramatically decreased lethality *in vivo*, due to being unable to bind to neurons with high affinity comparable to BoNT/A. Moreover, mutation of W985 reduced the toxicity of BoNT/A.

Research in the last few decades has greatly advanced understanding of the multi-phasic mechanism of action of BoNTs. This includes binding to SV2 (BoNT/A, /D, /E and /F) or synaptotagmin I/II (BoNT/B and /G) via H_CC_ subdomain, acceptor-mediated endocytosis, translocation by the H_N_, and cleavage of SNAREs by LC^1^. To determine the functional role of the conserved H_CN_, we first deleted this sub-domain from BoNT/A. Although its protease activity *in vitro* was not affected, rAΔH_CN_ failed to cleave the intra-neuronal substrate. This is due to loss of high-affinity binding to neurons, as quantified using the radio-iodinated toxins. Our results accord with a recent elegant report on the crystal structure of H_C_/A in complex with glycosylated human SV2C^23^. Several residues in H_CN_/A have been identified as being essential for binding and uptake of BoNT/A into neurons^23^. As expected, the neuronal membrane acceptors gave a higher affinity for ^125^I-rA than reported for glycosylated SV2C-L4 due to use of the full-length protein and endogenous gangliosides. These *in vitro* findings are reconcilable with the decreased ability of rA∆H_CN_ relative to rA to cause muscle weakening, and lethality in mice. We also show that rAΔH_CN_ at a high concentration can still bind but fails to enter into the cultured sensory neurons, suggesting that H_CN_ might additionally contribute to efficient internalisation. However, this requires further investigation.

An earlier study^25^ found that H_CN_ binds to PIPs which might allow the subsequent insertion of H_N_ into the vesicular membrane. Four positively-charged residues in H_CN_/A: Arg-892, Lys-896, Lys-902, Lys-910 were proposed to interact with PIP. However, mutating Arg892 and Lys896 or Lys902 and 910 to Ala in full-length of BoNT/A did not reduce its cleavage of SNAP-25 in cultured CGNs (Fig. S6). Interaction of H_CN_ in the H_C_ of BoNT mosaic serotype C/D with PG4 was also revealed by the crystal structure^26^, and 6 out of the 9 residues contacting PG4 are highly conserved between BoNT serotypes, A-F. Herein, mutating 5 out of 6 of these conserved residues within H_CN_ of BoNT/A only gave negligible or minor reduction in SNAP-25 cleavage. Similarly, pre-incubation of BoNT/A with an excess PG4 had no effect on its activity. Thus, our data do not seem to support the proposed roles of PIPs and PG4 in multi-phasic action of BoNT/A. Nevertheless, mutating W985 to L in full-length BoNT/A did not alter its binding to neurons, but caused over a 10-fold drop in the cleavage of intra-neuronal SNAP-25. Hence, our findings suggest that this residue, and probably others, may facilitate BoNT/A internalisation and/or translocation of the protease after initial acceptor binding.

To exploit the protease of BoNT/A for extended therapeutic applications, various approaches have involved replacement of the H_C_ domain of /A with moieties capable of targeting particular cell types in the nervous and endocrine systems to inhibit the release of neurotransmitters, hormones, neuropeptides and others^34^,^35^,^36^,^37^. An even more advanced hybrid protein was recombinantly created by inserting a modified growth hormone-releasing hormone (GHRH) domain into the loop region between LC and H_N_ of BoNT/D lacking the entire H_C_ domain^38^. Binding of this molecule to GHRH receptors *in vivo* leads to inhibition of growth hormone secretion in juvenile rats, eventually resulting in reduced body size, bone and mass acquisition^38^. However, the requirement of hundreds of micrograms of this recombinant protein per kilogram body weight restricts its clinical potential for treating acromegaly. Improving the potency of the latter and that of the above-mentioned therapeutics is necessary, and would be highly desirable for clinical purposes. Even with very potent BoNT complexes, repeated injections could lead to secondary treatment failure in some patients due to production of neutralizing antibodies^39^,^40^. To improve the potency of retargeted biotherapeutics, it would be helpful to retain the H_CN_ because, as shown herein, it not only contributes to acceptor binding but also to the internalisation/translocation steps. Of course, whether the targeting moiety and conserved H_CN_ can orchestrate retargeting of the variants into the desired cells await further testing.

Overall, our results reaffirm that each domain of BoNT acts in a ‘chaperone-like’ fashion for the others. Continued molecular definition of the function of each moiety, and yet to be identified factors, involved in its multi-step intoxication will not only shed insights into pathogenic mechanisms but also help in the design of more effective inhibitors to counteract botulism, as well as in the development of novel therapeutics for other hyper-secretory disorders.

## Materials and Methods

### Materials

Rabbit monoclonal anti-HA was purchased from Cell Signalling Technology (local distributor, Brennan and Company, Stillorgan, Ireland), and mouse monoclonal anti-VAMP2 from Synaptic System GmbH (Goettingen, Germany). Alexa Fluor 488 goat anti-mouse IgG, Alexa Fluor 488 and 568 goat anti-rabbit IgGs were obtained from Jackson ImmunoResearch (Hamburg, Germany) and Bio-Science (Dun Laoghaire, Ireland). PG4, standard cell culture medium and components were supplied by Sigma-Aldrich (Arklow, Ireland) and Bio-Sciences.

### Animals and ethics statement

Pups from rats (Sprague Dawley) bred in an approved Bio-Resource Unit at Dublin City University were used. The experiments, maintenance and care of the rodents complied with the European Communities (Amendment of Cruelty to Animals Act 1876) Regulations 2002 and 2005. Experimental procedures had been approved by the Research Ethics Committee of Dublin City University, and licenced by the Irish Health Products Regulatory Authority.

### Constructs for recombinant BoNT/A variants

Experiments involving recombinant BoNTs had been approved by the Biosafety Committee of Dublin City University, and the Environmental Protection Agency of Ireland. To obtain rAΔH_CN_, nucleotides encoding residues Ile_874_-Q_1091_ were deleted with insertion of GGC GGT by inverted PCR, using a previously-reported pET29a-BoNT/A construct as template^19^. The resultant PCR products were self-ligated by T4 ligase and transformed into Top10 competent cells for screening of positive clones. In order to engineer rA-HA and rAΔH_CN_-HA, a short nucleotide sequence encoding HA tag (YPYDVPDYA) was inserted before the thrombin recognition site located in the loop region of rA and rAΔH_CN_. Constructs encoding BoNT/A with one or more mutated residues in the H_CN_ region were made by site-direct mutagenesis.

### Production of recombinant toxins

After verifying all of the above constructs, plasmids were transformed into *E. coli* BL21.DE3 for expression, using an auto-induction medium^41^. Recombinant proteins were purified by IMAC on Talon resin. rA, rAΔH_CN_ and their fusions with a HA tag were further purified by anion-exchange chromatography, following the protocol established for rA^19^. To activate the toxin, SC was incubated with thrombin (1 mg/1 unit of thrombin) at 22 °C for 1 h before adding phenylmethylsulfonyl fluoride to 1 mM concentration to stop the reaction. Protein concentration was quantified by Bradford reagent.

### GST-SV2C-L4 pull down assay

GST-tagged SV2C–L4_(454–580)_ protein (100 μg) was incubated with 50 μl of glutathione Sepharose (Fisher Scientific, Ballycoolin, Ireland) for 1 h at 4 °C. After washing with 1 ml of binding buffer^30^,^32^, resin was incubated with different concentrations of rA, rAΔH_CN_ or rA(W_985_L) DC in binding buffer. After washing 3 times with 1 ml of binding buffer, bound proteins were eluted by LDS sample buffer containing DTT with a final concentration of 50 mM. The reduced samples were analyzed by SDS-PAGE followed by Western blotting, using antibodies against LC/A or GST.

### Measurements of protease activity, lethality and neuromuscular paralysis of the generated BoNTs

Proteolytic activities of rA, rAΔH_CN_ and rA(W_985_L) were determined using a recombinant model substrate, green fluorescent protein (GFP)-SNAP25-C_73_-His_6_^27^. Briefly, the toxins were diluted to 50 nM in HBS-20 [20 mM HEPES, 100 mM NaCl, pH 7.4; 10 μg/ml bovine serum albumin (BSA); 5 mM DTT and 10 μM ZnCl_2_]. This mixture was incubated at 37 °C for 30 min before a 2-fold serial dilution in HBS-20 and mixing with an equal volume of substrate (1 mg/ml). After further incubation for 30 min at 37 °C, reactions were stopped by adding ice-cold LDS sample buffer. Intact substrate and the larger BoNT-cleaved product were separated by SDS-PAGE (NuPAGE 12% precast Bis-Tris gel) and visualized by Coomassie staining.

The specific lethalities of rA and rAΔH_CN_ were measured using a mouse lethality assay^28^. Briefly, this involved intraperitoneal injection into Tyler’s Ordinary mice of several amounts of each toxin: 1–10 pg for rA, and 1–1000 ng for rAΔH_CN_. The observed number of death within 5 days indicated the approximate lethalities; then, the assay was repeated by injecting 4 animals each with doses close to the expected LD_50_ (5–8 pg of rA, 100–500 ng of rAΔH_CN_). The dose which killed half of each group was taken as a minimal LD_50_ value. Their relative abilities to induce neuromuscular paralysis were determined by the DAS values^29^ recorded over time, following injection of the indicated amounts in 5 μl into the right gastrocnemius muscle of groups of 7 mice.

### Primary culture of rat CGNs, TGNs and their treatment with BoNTs

Isolation and culture of CGNs and TGNs from 4–7 day old rats have been described previously^6^,^28^. Cultured neurons at 10–14 days *in vitro* (DIV) were incubated with various concentrations of each toxin in culture medium for 24 h before harvesting in LDS sample buffer. In one case, rat CGNs were incubated with BoNT/A or W985L mutant in low potassium (LK) buffer (mM: 20 HEPES, 120 NaCl, 5 KCl, 2 MgCl_2_, 1.3 CaCl_2_, and 5 glucose) for 8 min as described in ref. 16. After removing the unbound toxin by washes with DMEM, cells were further cultured in fresh medium for 5 h before harvesting in LDS sample buffer. Intact and BoNT-cleaved SNAP-25 were separated by SDS-PAGE (NuPAGE 12% precast Bis-Tris gel) and visualised by Western blotting, using an antibody recognizing both cleaved and intact forms. The proportion of intact SNAP-25 remaining was calculated relative to the total (cleaved plus remaining intact), using image J software analysis of digitised images.

### Radio-iodination of BoNTs and their binding to CGNs

rA, rAΔH_CN_ and rA(W_985_L) were iodinated using sodium ^125^Iodine and a previously-described chloramine-T method with slight modification^33^. Briefly, each BoNT (40 μg in 40 μl) was added to 1 mCi (10 μl) of carrier free Na ^125^I and derivatisation initiated by adding chloramine-T (5 μl of 2 mM to a final concentration of 0.22 mM). The reaction was quenched after 40 s by addition of an excess of L-tyrosine (25 μl of 1 mg/ml in 0.1 M sodium phosphate buffer pH 7.4 containing 150 mM NaCl). Free ^125^I and tyrosine were separated from the ^125^I-labelled toxin by gel filtration, using a PD-10 column equilibrated with the latter buffer. ^125^I-toxins were stored at 4 °C in the presence of gelatin to a final concentration of 0.25% (w/v), following removal of aliquots to determine their specific radioactivities.

Cultured CGNs were suspended in PBS containing 1 mg/ml BSA. Aliquots (50–150 μg) were incubated with increasing concentrations of the ^125^I-labelled toxin for 1 hour at 4 °C, or together with its unlabelled counterpart at ≥100-fold molar excess over the highest concentration of ^125^I-toxin used. Binding was terminated by centrifugation (9,000× g for 2 min) and resuspension in ice-cold PBS, after which pellets were washed two further times as described prior to γ-counting.

### Immuno-fluorescence staining

Rat TGNs grown on coverslips or μ -Slide 8-well Ibidi chambers (Ibidi GmbH, Martinsried, Germany) at 7 DIV were incubated with 100 nM rA, rA-HA or rAΔH_CN_-HA for 24 h at 37 °C in culture medium. Cells were then washed three times with PBS before fixation with 3.7% paraformadehyde in PBS for 30 minutes. The samples were then washed thrice with PBS, followed by permeabilisation for 5 minutes with 0.2% Triton X-100 in PBS before blocking with 1% BSA in PBS for 1 hour. A pair of primary antibodies [rabbit monoclonal anti-HA (1:1600) and mouse monoclonal anti-VAMP2 (1:1000)] were applied in the blocking solution for 1 hour at room temperature. Washed samples were incubated with fluorescent secondary antibodies (Alexa Fluor 488 goat anti-mouse IgG and Alexa Fluor 568 goat anti-rabbit IgG) for 1 hour. After five washes with PBS, the samples were then mounted with Vectashield (Vector Laboratories) and fluorescent images captured with an inverted Zeiss LSM 710 confocal microscope (Carl Zeiss Microimaging) using Zen2008 software (Universal Imaging, Göttingen).

### Statistical analysis

Probability values were determined with the use of Student’s unpaired two-tailed t-test or two-way ANOVA followed by Bonferroni post hoc test by GraphPad Prism software, as specified in figure legends. Values of P < 0.05 were considered significant.

## Additional Information

**How to cite this article**: Wang, J. *et al*. Neuronal entry and high neurotoxicity of botulinum neurotoxin A require its N-terminal binding sub-domain. *Sci. Rep.* **7**, 44474; doi: 10.1038/srep44474 (2017).

**Publisher's note:** Springer Nature remains neutral with regard to jurisdictional claims in published maps and institutional affiliations.

## Supplementary Material

Supplementary Information
