# Supplementary material for: Neuronal entry and high neurotoxicity of botulinum neurotoxin A require its N-terminal binding sub-domain
Source: Sci Rep. 2017 Mar 15;7:44474. doi: 10.1038/srep44474 (PMC5353748; doi:10.1038/srep44474)
Supplement: Supplementary Information [file srep44474-s1.pdf]

Supplementary Information

Neuronal entry and high neurotoxicity of botulinum neurotoxin A require its N-terminal binding sub-domain

Jiafu Wang, Jianghui Meng, Marc Nugent, Minhong Tang, J. Oliver Dolly

International Centre for Neurotherapeutics, Dublin City University, Glasnevin, Dublin 9, Ireland.

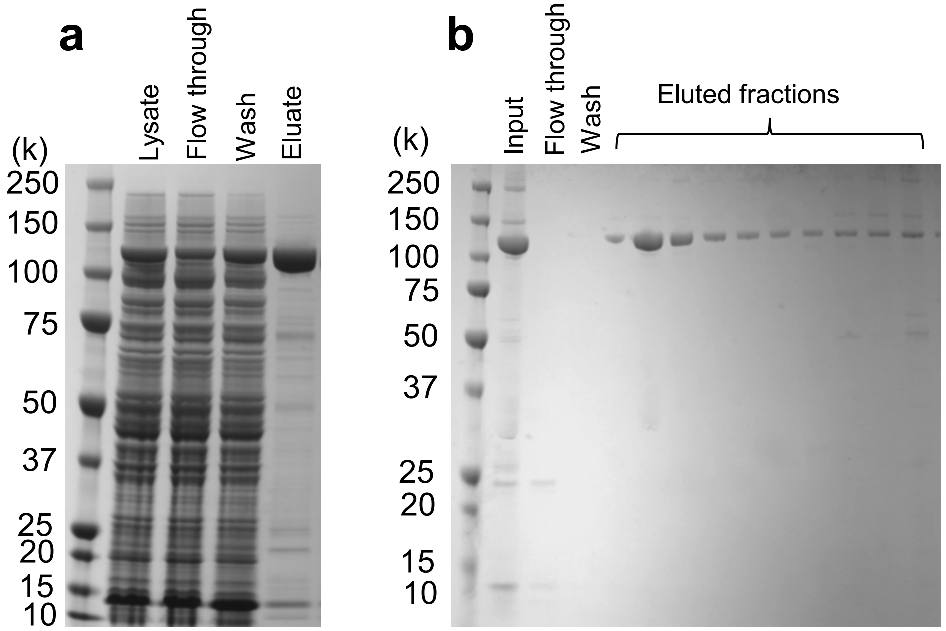

Figure S1. Purification of rA∆H_{CN} by IMAC followed by anion-exchange chromatography. (**a**) rA∆H_{CN} expressed in *E. coli* was purified by IMAC; aliquots were subjected to reducing SDS-PAGE followed by Coomassie staining. (**b**) Pooled eluate from IMAC was buffer exchanged into 50 mM Tris-HCl buffer (pH 8.1) using Sephadex G-25 and loaded onto a Resource Q column; after washing with 30 mM NaCl, a stepwise gradient up to 1 M NaCl in the above buffer was applied. Aliquots were analysed as in (**a**).

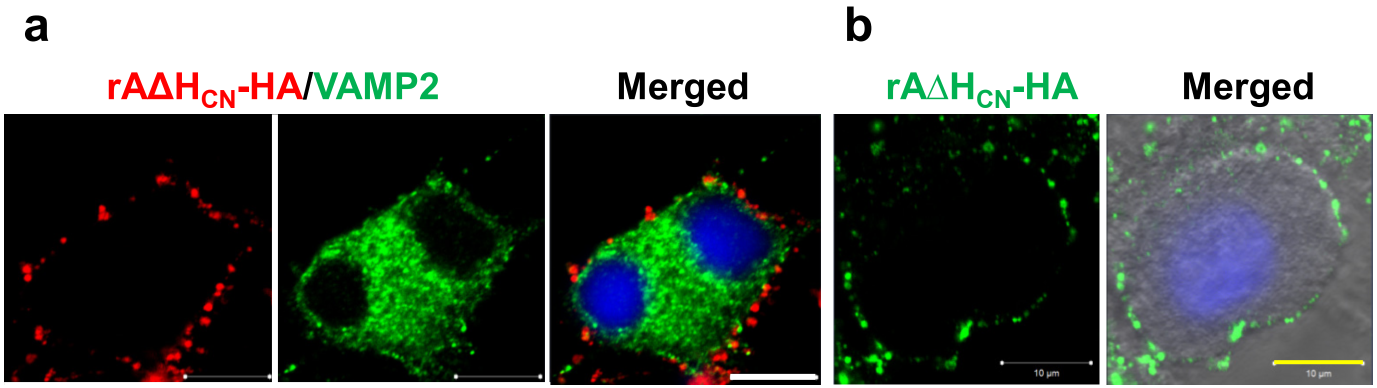

Figure S2. A majority of rAΔH_{CN}-HA remained on the plasmalemma even after 24 h exposure to cultured sensory neurons. Rat TGNs on coverslips at 7 DIV were incubated with 100 nM of rAΔH_{CN}-HA for 24h at 37°C in culture medium. Washed cells were fixed with 3.7% paraformadehyde in PBS. The samples were then washed three times with PBS, followed by incubation with (**a**) or without (**b**) 0.2% Triton X-100 in PBS for 5 min. before blocking with 1% BSA in PBS for 1 hour. (**a**) A pair of primary antibodies [rabbit monoclonal anti-HA (1:1600) and mouse monoclonal anti-VAMP2 (1:1000)] were applied in the blocking solution for 1h at room temperature. Washed samples were incubated with fluorescent secondary antibodies (Alexa Fluor 488 goat anti-mouse IgG and Alexa Fluor 568 goat anti-rabbit IgG) for 1 h. In panel **b**, non-permeabilised samples were incubated with anti-HA primary antibody, followed by Alexa Fluor 488 goat anti-rabbit IgG. Nuclei were counter-stained with DAPI (4',6-diamidino-2-phenylindole). Fluorescent images were captured with a Zeiss LSM 710 confocal microscope. Bars, 10 µm.

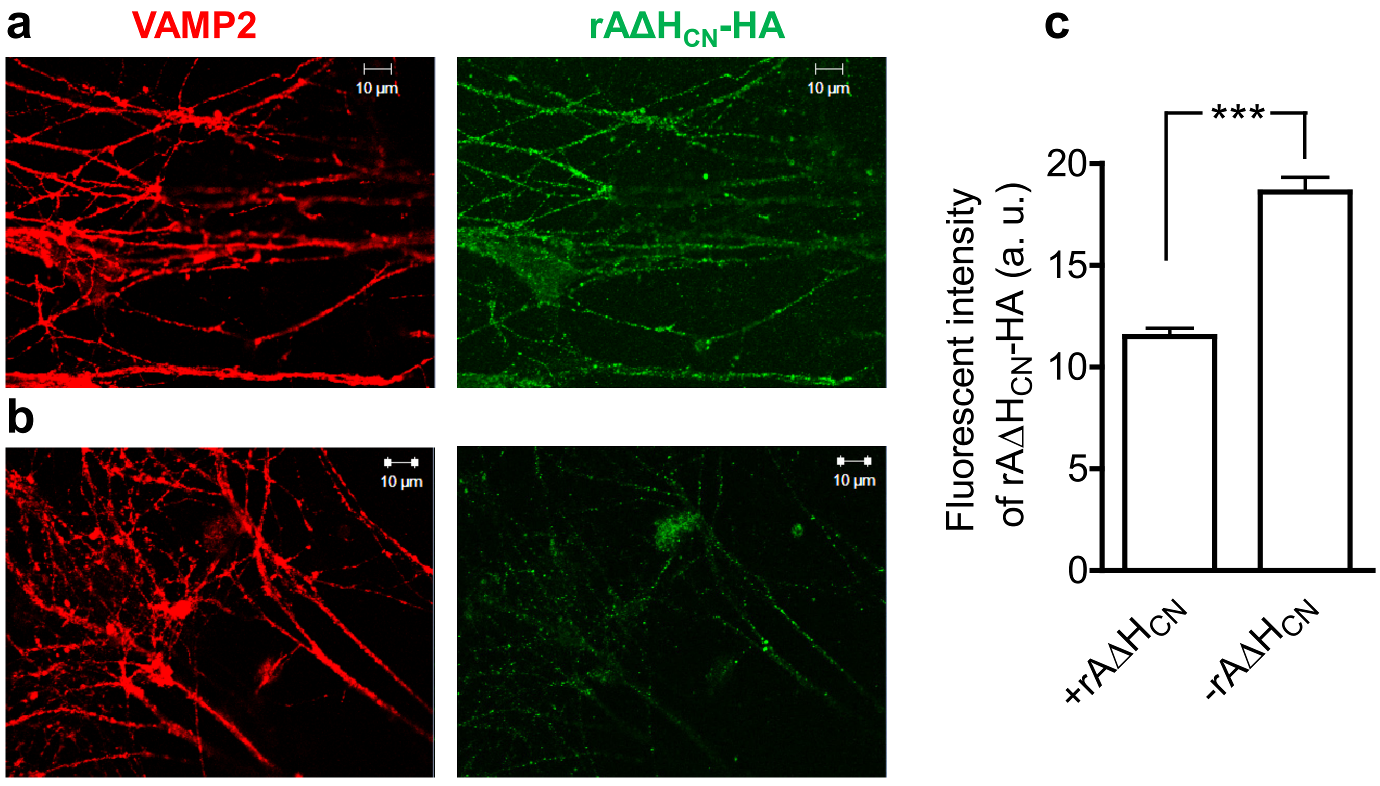

Figure S3. Binding of HA tagged rAΔH$_{CN}$ to sensory neurons was reduced by an excess of untagged rAΔH$_{CN}$. Rat TGNs on Ibidi culture chambers at ~7 DIV were incubated with 100 nM of rAΔH$_{CN}$-HA alone in HK buffer **(a)** or together with 1 μM untagged rAΔH$_{CN}$ SC (**b**) for 10 min. at 37°C. Cells were than washed three times with PBS before fixing with 3.7% paraformadehyde in PBS. Washed samples were permeabilised by incubation with 0.2% Triton X-100 in PBS for 5 min. before blocking with 1% BSA in PBS for 1 h. Rabbit monoclonal anti-HA and mouse monoclonal anti-VAMP2 antibodies were applied in the blocking solution for 1 h at room temperature. Washed samples were incubated with fluorescent secondary antibodies (Alexa Fluor 568 goat anti-mouse IgG and Alexa Fluor 488 goat anti-rabbit IgG) for 1 h. Ffluorescent images were captured with a Zeiss LSM 710 confocal microscope. Bars, 10 μm. (**c**) Mean fluorescence intensity of rAΔH$_{CN}$-HA in arbitrary units from **a** and **b** from ~20 randomly selected images from 3 independent cultures. Data plotted are the means ± S.E.M., ***< 0.001 (the unpaired two-tailed Student's t-test).

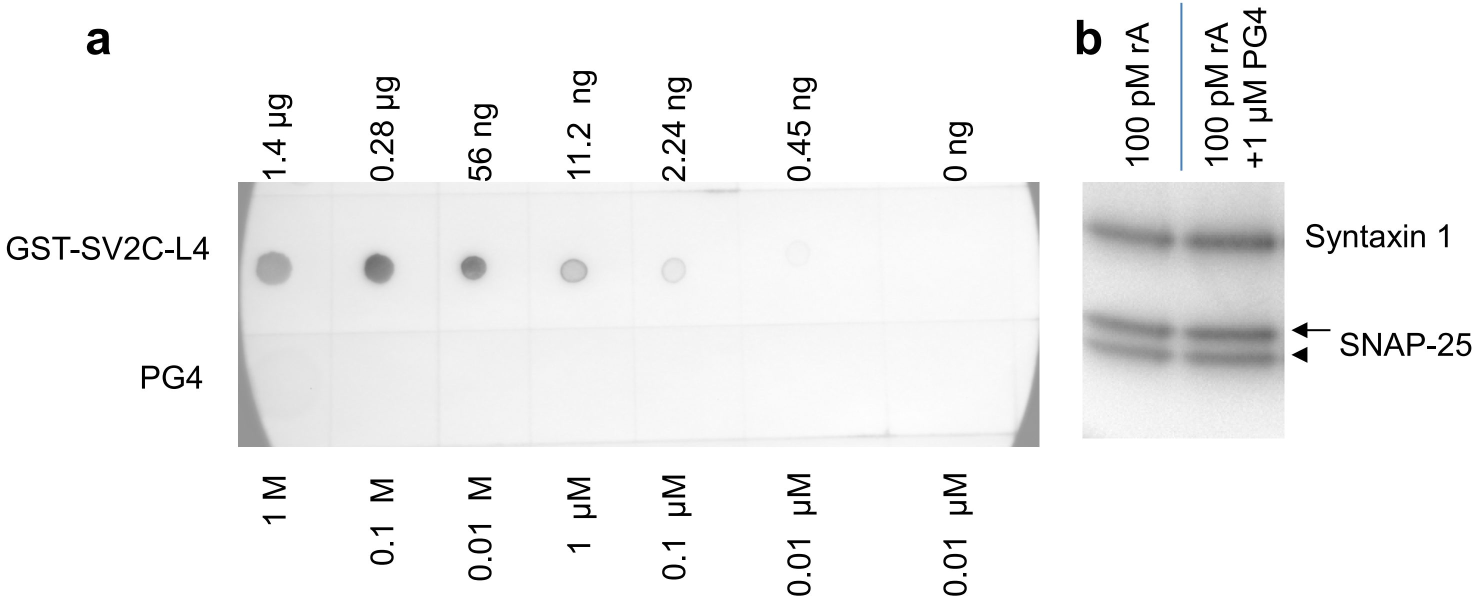

Figure S4. Probing the interaction between PG4 and rA. (**a**) After spotting 2 µl of serially-diluted GST-SV2C-L4 and PG4 on nitrocellulose membrane, the dried membrane was blocked with 5% BSA in TBST (tris-buffered saline, 0.1% Tween 20) before being incubated with 10 ml of 0.15 µg/ml of BoTIM/A for 1h. The washed membrane was further incubated with rabbit anti-LCA antibody for 1 h followed by horse radish peroxidase (HRP) labelled donkey anti-rabbit secondary antibodies before developing with enhanced chemiluminescence reagents. (**b**) 100 pM rA with or without 1 µM PG4 in HK depolarisation buffer were incubated at 37°C for 20 mins before intoxicating CGNs for 8 mins at 37°C. Cells were then washed three times before further culturing in medium for 5 h. Cleavage of SNAP-25 was probed by Western blotting with an antibody recognising both intact (arrow) and cleaved SNAP-25 (arrowhead). Syntaxin 1 was blotted as internal loading control.

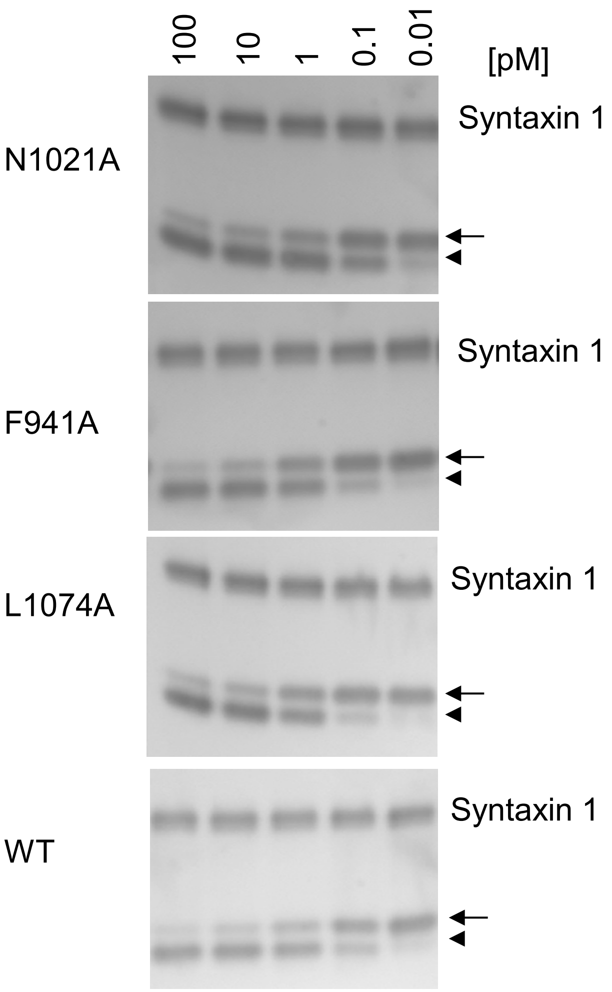

Figure S5. Effects of single amino acid mutation in H_{CN} region of BoNT/A on cleavage of SNAP-25 in rat CGNs. After 24h exposure of the cultured CGNs to the different concentrations in culture medium of the toxin variants shown, the samples were subjected to SDS-PAGE followed by Western blotting with an antibody recognizing both intact (arrows) and cleaved SNAP-25 (arrowheads). Syntaxin 1 was probed as internal loading control.

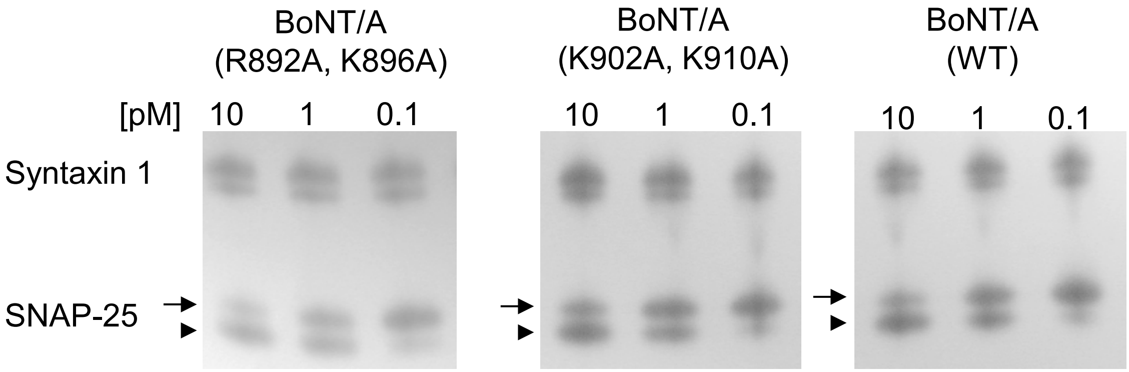

Figure S6. Mutation of R892 and K896 or K902 and K910 in H_{CN} of BoNT/A to alanines did not reduce its potency in cleaving SNAP-25 in rat CGNs. CGNs were exposed to the different concentrations of BoNT/A or its mutated variants (as indicated) for 16h before being harvested in LDS-sample buffer. Cleavage of SNAP-25 was probed by Western blotting with an antibody recognizing both intact (arrows) and cleaved SNAP-25 (arrowheads). Syntaxin 1 was used as internal loading control.
